# Measuring the Effect of Examiner Variability in a Multiple-Circuit Objective Structured Clinical Examination (OSCE)

**DOI:** 10.1097/ACM.0000000000004028

**Published:** 2021-03-02

**Authors:** Peter Yeates, Alice Moult, Natalie Cope, Gareth McCray, Eleftheria Xilas, Tom Lovelock, Nicholas Vaughan, Dan Daw, Richard Fuller, Robert K. (Bob) McKinley

**Affiliations:** 1 **P. Yeates** is a senior lecturer in medical education research, School of Medicine, Keele University, Keele, Staffordshire, and a consultant in acute and respiratory medicine, Fairfield General Hospital, Pennine Acute Hospitals, NHS Trust, Bury, Lancashire, United Kingdom; ORCID: https://orcid.org/0000-0001-6316-4051.; 2 **A. Moult** is a research assistant in medical education, School of Medicine, Keele University, Keele, Staffordshire, United Kingdom; ORCID: https://orcid.org/0000-0002-9424-5660.; 3 **N. Cope** is a lecturer in clinical education (psychometrics), School of Medicine, Keele University, Keele, Staffordshire, United Kingdom.; 4 **G. McCray** is a researcher, School of Primary, Community and Social Care, Keele University, Keele, Staffordshire, United Kingdom.; 5 **E. Xilas** is a foundation year 1 doctor and recent graduate, School of Medicine, Keele University, Keele, Staffordshire, United Kingdom.; 6 **T. Lovelock** is an information technology services manager, Faculty of Medicine & Health Sciences, Keele University, Keele, Staffordshire, United Kingdom.; 7 **N. Vaughan** is a senior application developer, directorate of digital strategy and information technology services, Keele University, Keele, Staffordshire, United Kingdom.; 8 **D. Daw** is an information technology systems development engineer, School of Medicine, Keele University, Keele, Staffordshire, United Kingdom.; 9 **R. Fuller** is deputy dean, School of Medicine, University of Liverpool, Liverpool, United Kingdom; ORCID: https://orcid.org/0000-0001-7965-4864.; 10 **R.K. McKinley** is an emeritus professor of education in general practice, School of Medicine, Keele University, Keele, Staffordshire, United Kingdom; ORCID: https://orcid.org/0000-0002-3684-3435.

## Abstract

Ensuring that examiners in different parallel circuits of objective structured clinical examinations (OSCEs) judge to the same standard is critical to the chain of validity. Recent work suggests examiner-cohort (i.e., the particular group of examiners) could significantly alter outcomes for some candidates. Despite this, examiner-cohort effects are rarely examined since fully nested data (i.e., no crossover between the students judged by different examiner groups) limit comparisons. In this study, the authors aim to replicate and further develop a novel method called Video-based Examiner Score Comparison and Adjustment (VESCA), so it can be used to enhance quality assurance of distributed or national OSCEs.

**Method:**

In 2019, 6 volunteer students were filmed on 12 stations in a summative OSCE. In addition to examining live student performances, examiners from 8 separate examiner-cohorts scored the pool of video performances. Examiners scored videos specific to their station. Video scores linked otherwise fully nested data, enabling comparisons by Many Facet Rasch Modeling. Authors compared and adjusted for examiner-cohort effects. They also compared examiners’ scores when videos were embedded (interspersed between live students during the OSCE) or judged later via the Internet.

**Results:**

Having accounted for differences in students’ ability, different examiner-cohort scores for the same ability of student ranged from 18.57 of 27 (68.8%) to 20.49 (75.9%), Cohen’s *d* = 1.3. Score adjustment changed the pass/fail classification for up to 16% of students depending on the modeled cut score. Internet and embedded video scoring showed no difference in mean scores or variability. Examiners’ accuracy did not deteriorate over the 3-week Internet scoring period.

**Conclusions:**

Examiner-cohorts produced a replicable, significant influence on OSCE scores that was unaccounted for by typical assessment psychometrics. VESCA offers a promising means to enhance validity and fairness in distributed OSCEs or national exams. Internet-based scoring may enhance VESCA’s feasibility.

## Abstract 2

Supplemental Digital Content is available in the text.

Despite innovations in assessment that claim greater authenticity and greater influence on learning, ^1–3^ objective structured clinical examinations (OSCEs) ^4^ remain a cornerstone of most assessment systems in medical education. They remain in wide use because of their apparent ability to ensure consistency and fairness, ^5,6^ and, in turn, assure patients and the public that resulting licencing decisions are warranted. ^7,8^ Consequently, whilst the validity of OSCEs is evidenced by a chain including station design, blue-printing, rating scale characteristics, and examiner training, thoroughly understanding examiner variability remains critical to the resulting validity argument. ^9^ This report describes developments of a novel method to enhance the understanding of examiner variability.

Part of the original validity argument for OSCEs relied on the premise that all students meet all examiners as they rotate around the OSCE circuit, a process intended to negate examiner differences. ^10^ As the number of students has increased, many institutions have begun to run multiple, parallel, simultaneous OSCE circuits—sometimes distributed across different geographical sites with different examiners in each circuit. As each student encounters only a single “examiner-cohort” ^11^ (i.e., the particular group of examiners allocated to a specific circuit and examination time), any collective difference in judgments across examiner-cohorts that influences pass/fail decisions could substantially challenge fairness and validity of the exam.

Psychometric analyses of OSCEs typically ignore the influence of examiner-cohorts because fully nested OSCE designs (no crossover exists among students seen by different groups of examiners) make this particular factor impossible to analyze. Prior work has suggested that differences in examiner-cohorts account for 4.4% of the difference across distributed locations in a U.K. medical school, ^11^ that standardized patient (SP)-raters at different sites account for up to 15.7% of score variance across 21 sites in the United States, ^12^ and that SP-raters at 6 different sites contribute between 2.0% and 17.1% of score variance on the Medical Council of Canada National Assessment Collaboration Examination for international medical graduates. ^13^ These findings suggest potentially significant effects due to assessor scoring variance at an international level.

Whilst these studies hinted at the potential for important influences on distributed or even national licencing exams, all 3 were methodologically limited by the fully nested examiner design already described. ^5,14^ To explain, these 3 studies worked on an implicit assumption that student ability is similar on average across sites (i.e., as if a model of true randomization to sites and circuits had been employed). In 2019, Yeates and colleagues ^15^ reported pilot work on a novel methodology called Video-based Examiner Score Comparison and Adjustment (VESCA). In addition to making in-person (or “live”) judgments of students, all examiner-cohorts were asked to score a common pool of videos of students’ OSCE performances, thereby creating partial crossing and, in turn, enabling analysis of otherwise fully nested examiner-cohort effects. This pilot work demonstrated a large difference (Cohen’s *d* = 1.06) across the average ratings of some examiner-cohorts. If replicated in distributed summative OSCEs or in national licencing exams, this effect could substantially influence outcomes for many candidates, thereby challenging the validity of the assessment, with consequences for candidates, patients, and institutions as a result of potentially “incorrect” pass/fail decisions.

The primary aim of this study was to replicate the effects reported by Yeates and colleagues ^15^ on a different sample of students and examiners by using VESCA to compare and adjust for the influence of examiner-cohorts on students’ scores. We also addressed 2 secondary aims: first, to extend Yeates and colleagues’ work ^15^ by investigating how score adjustments influence students’ classification for a range of different cut scores (rather than the single arbitrary cut score previously reported), and second, to expand upon their methods by comparing the influence of different video-based scoring methods on examiners’ scores and participation rates. In so doing, we aimed to further develop VESCA with the goal of enhancing the quality assurance of distributed OSCEs or national licencing exams.

## Method

We used VESCA ^15^ to compare and adjust for the influence of different examiner-cohorts within a multicircuit OSCE. Theoretically, this intervention considers “examiners as fallible” ^16^ and seeks to assess residual examiner differences after faculty development, training, and benchmarking have all been used to maximal effect to aid standardization. VESCA uses 3 sequential processes:

1. A small sample of candidates are unobtrusively videoed whilst performing on all stations within a real OSCE.
2. Examiners mark the live student performances and are invited to score common station-specific comparator videos. Examiners from each cohort collectively score the same videos, linking scores across otherwise fully nested groups of examiners.
3. Statistical analyses are used to compare and adjust for examiner influences.

To increase the linkage across examiner-cohorts, we asked examiners to score 4 videos of the station they examined, rather than the 2 videos scored previously. ^15^ Four videos represented a balance between increased linkage and the time demands of an intervention we hope can be used in routine OSCEs.

### Setting

**Figure 1:**
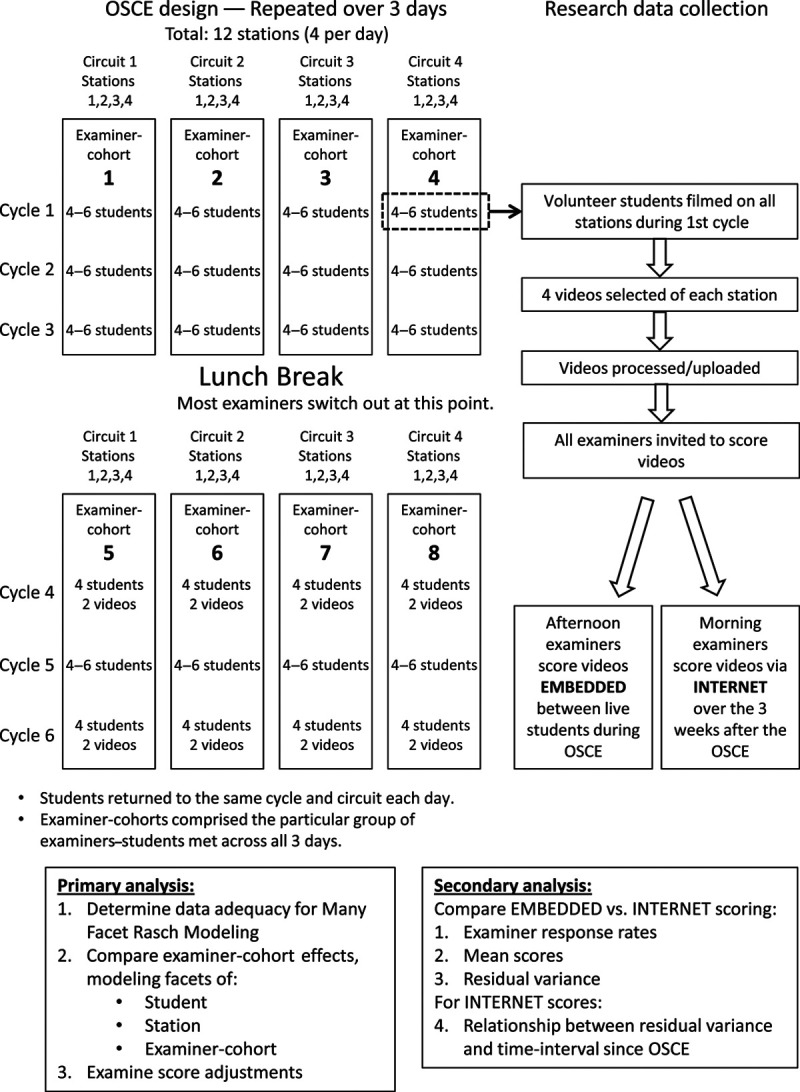
Schematic overview of study design and research data collection for an investigation to measure the effect of examiner variability in a multiple-circuit objective structured clinical examination (OSCE), 2019, School of Medicine, Keele University, United Kingdom.

In April and May 2019, we gathered data from the year 3 summative OSCE in the undergraduate medicine course at the School of Medicine, Keele University. Year 3 is the first predominantly clinical year of the 5-year program. The OSCE comprised 12 stations, including consultation, physical examination, and procedural skills. Each station integrated a range of communication, diagnostic reasoning, and practical (technical/procedural) skills. Cases were portrayed by scripted simulated or real patients. Students were assessed by trained examiners, all of whom were either medical doctors or clinical skills tutors. Examiners’ training comprised a video-based benchmarking exercise and a pre-OSCE briefing on the scoring format. Performances were scored on Keele’s domain-based rating scale called GeCoS, ^17^ which provides a score out of 27 points for each station (see Supplemental Digital Appendix 1 at http://links.lww.com/ACADMED/B74 for an example). The OSCE was conducted over 3 days, with 4 stations per day. All students attended on all 3 days to complete all 12 stations. Four separate parallel circuits of the OSCE were conducted, repeated in the morning and afternoon with different students and occasionally overlapping examiners. This design produced 8 separate examiner-cohorts. See Figure 1 for a schematic of the OSCE and research design.

### Population, recruitment, and sampling

Participation in the study was voluntary for all individuals. All students, examiners, and simulated or volunteer real patients in the OSCE were eligible and were invited via email to participate. Filming was conducted in a single circuit during the first rotation of each day (see Figure 1), and the maximum capacity was 6 students. Filming volunteers were allocated in order of agreement. Written consent was obtained from all participants, and participants retained the right to withdraw up until the end of filming. Ethical approval was granted by Keele Ethical Review Panel (ERP2413).

### Filming OSCE performances

Performances on each station were filmed simultaneously by 2 unobtrusively positioned closed-circuit TV cameras (one wide angle, one zoomed; ReoLink RLC-423, Wan Chai, Hong Kong). The OSCE otherwise proceeded exactly as normal for the students who were filmed. The lead author (P.Y.) selected camera angles a priori to ensure adequate images of key components of the task. Videos in which key performance elements were obscured were omitted, and the first 4 available videos with clear views were selected for each station for video-based scoring by examiners. Videos were processed to give simultaneous side-by-side views of the procedural and physical examination stations, whilst (to minimize digital imaging processing requirements) a single view was selected in editing for the consultation (history, clinical reasoning, or patient management) stations.

### Video scoring by examiners

We compared 2 separate methods of video-scoring by examiners, which we termed (1) “Internet” and (2) “embedded.”

#### Internet examiners.

All examiners from the morning session of each OSCE day were invited via email to participate by visiting a password-protected website. Those who agreed provided consent electronically and scored 4 videos specific to the station they had already examined. The website provided a downloadable PDF of the station-specific examiner information and scoring rubric. Internet examiners entered scores for these video performances via a form on the website. Participants had 3 weeks from the OSCE to complete scoring. As a result, Internet scoring was highly flexible for the examiners. Further, Internet scoring avoided additional time demands during the OSCE.

#### Embedded examiners.

Examiners in the afternoon session of each OSCE day were invited to score the same 4 station-specific videos “embedded” within their OSCE examining circuit. To achieve this, 4 gaps were created within the rotation of students in the afternoon, and participating examiners were provided with tablet computers to view and score performances. Whilst this scheduling ensured that videos were scored in close proximity to live judgments, it required an additional 40 minutes per OSCE session.

In both the Internet and embedded conditions, examiners were asked to avoid extraneous distractions and to give videos their full attention, scoring them as they would in the OSCE. To ensure similarity with live observations, examiners were asked to watch each video once only and to neither pause nor rewind. As in Yeates and colleagues (2019), ^15^ examiners who judged the filmed performances live also scored them again via video to enable comparison of their video and live scores. We aligned data from live and video performances before analyses.

### Analysis

We addressed our primary aim, to compare and adjust the influence of examiner-cohorts on students’ scores, using Many Facet Rasch Modeling (MFRM). ^18^ We used ratings on the 27-point rating scale as the dependent variable and modeled the following facets: (1) student, (2) examiner-cohort, and (3) station. Analyses were conducted using FACETS v3.82.3 (2019, Winsteps, Beaverton, Oregon). We performed several analyses to ensure the validity of the MFRM analysis. First, we examined for bias between live and video scores within the subset of performances for which both were available, using a Bland–Altman plot. ^19^ We performed this analysis using the BlandAltmanLeh package in RStudio: Integrated Development for R (RStudio version 1.2, running R version 3.6.3, 2020; RStudio, Inc., Boston, Massachusetts). Next, we examined how well data met the assumptions of MFRM; that is, we examined the progression of Rasch-Andrich thresholds ^20^ for each rating scale category to determine whether scores were ordinal and to determine the fit of data to the model for each facet, using Linacre’s fit criteria (i.e., mean square infit/outfit 0.5–1.5 = good fit ^21^). Next, as MFRM requires unidimensional data, we performed principal component analysis (PCA) on model residuals to exclude any additional dimensions (or factors) in the data.

Continuing with our primary aim, we used adjusted scores supplied by FACETS to calculate each student’s score adjustments (i.e., that student’s raw average score minus their adjusted average score). Using these data, we calculated the percentage of students whose scores were adjusted by an amount equal to or greater than 0.5 standard deviations (SDs) of the distribution of students’ ability.

To understand the potential effect of score adjustment on students’ pass/fail classifications, we examined the proportion of students whose adjusted score placed them in a different pass/fail class than their raw score had placed them in. As any such analysis is likely to depend on the proportion of students who fail based on their raw score, and the failure rates of OSCEs vary between exams, we modeled the effect of changing the classification for OSCEs with a range of failure rates. We modeled all possible cut scores at intervals of 0.1 score points—from 16.0 of 27 (59.3%), at which 0% of students failed on their raw score, to 19.0 of 27 (70.4%), at which 33.4% of 113 students failed on their raw score. We then calculated the percentage of students whose classification changed for each of these cut scores (from pass to fail, or from fail to pass).

Moving to our secondary aims, we determined the feasibility and influence of the 2 methods of video scoring (Internet vs embedded). First, we examined response rates for both methods. Next, we compared mean scores and error variances between the 2 modalities. Error variances compared whether examiners’ scores showed greater variability in one condition than the other. To examine error variances, we used a mixed-effects regression model, which corrected for student ability (random effect), station difficulty (fixed effect), and modality (Internet vs embedded, fixed effect) using the package “lme4” within RStudio: Integrated Development for R (RStudio version 1.2, running R version 3.6.3, 2020; RStudio, Inc., Boston, Massachusetts). We examined beta coefficients for each modality to determine whether scores differed, and we used an F test to compare error variances between modalities.

For the Internet scoring condition, we investigated whether examiners’ scores varied more from the mean (i.e., became less accurate) as the interval of time increased between the OSCE and the examiner scoring their videos. To do this, we first calculated the difference between each score given to each video by each examiner compared with the mean of the scores given to that video by all examiners. We expressed this as a mean absolute error (MAE), by calculating:


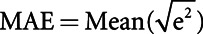


where *e* = an examiner’s score for a video − mean of all examiners’ scores for that video.

We then examined the relationship between videos’ MAE and the hours elapsed since the OSCE, using linear regression. We conducted this analysis using IBM SPSS Statistics for Windows v21.0 (IBM Corporation, Armonk, New York).

## Results

### Summary data

One hundred thirteen students completed the OSCE. Eight students volunteered for filming; only the first 6 were included in filming because of capacity limitations. No filmed volunteers withdrew, and all 12 stations were included. The unadjusted average ability for the cohort of 113 students across all 12 stations was normally distributed, ranging from 16.0 points out of 27 (59.3%) to 22.7 points (84.1%). The mean score was 19.5 of 27 (72.2%), and the SD was 1.43 (5.3%). By comparison, the unadjusted average ability of the 6 videoed students ranged from 17.2 of 27 (63.7%) to 21.3 (78.9%), indicating their ability was broadly representative of the overall cohort. No other details of filmed students were collected. Of 96 examiners, 73 (76.0%) chose to participate in video scoring. Participating and nonparticipating examiners’ scores for live performances were similar (participating examiners’ mean score = 19.4, SD = 3.8; nonparticipating examiners’ mean score = 19.9, SD = 3.2). Video scores comprised 17.7% of all data.

### Ensuring data adequacy for MFRM analysis

**Figure 2:**
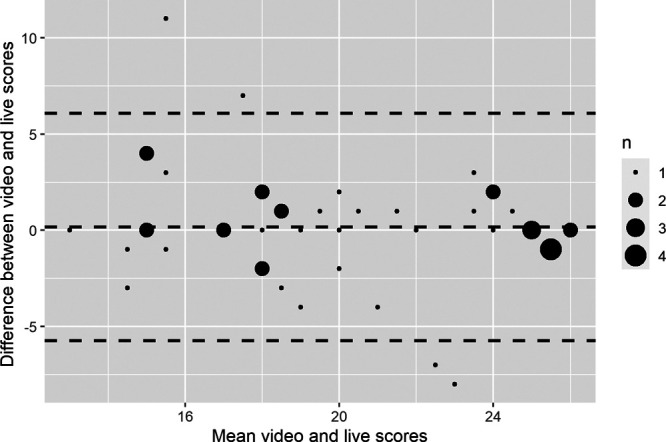
Bland–Altman plot indicating the difference between live and video scores (out of 27, vertical axis) plotted against the mean scores given to video and live versions of each performance (out of 27, horizontal axis) for the subset of examiners who judged the same performances by both methods. Dashed horizontal lines indicate the mean difference between the 2 measures and 1.96 standard deviations from the mean difference; n = the number of observations, as indicated by each dot.

Comparison of live and video scores for the subset of examiners who scored the same students by both methods indicated no systematic difference between the scores produced by the 2 modalities (average difference 0.16, 95% confidence interval [CI] −1.52 to 1.85; see Figure 2). Rasch-Andrich threshold progression for the 27-point rating scale showed disordered thresholds for points 8 and 11 on the rating scale (i.e., point 8 received a lower logit value than point 7, rather than the expected increase in logit value, and point 11 received a lower logit value than point 10). Cumulatively, these accounted for less than 2% of observations. All remaining logit values increased progressively, suggesting the scale progressed in the expected order to a sufficient degree to avoid compromising the analysis.

Data fitted the MFRM well. All examiner-cohorts and stations showed excellent or good fit, whilst 99% of students showed good model fit or acceptable model fit (either overfit or mild underfit). One student showed potentially degrading underfit. That student’s data had the potential to distort the model in a manner which, at least theoretically, might bias the estimates for other students. Omitting this student altered all students’ collective ability estimates by < 0.1%, so we continued with the complete dataset. Fit parameters are displayed in Table 1. PCA of residuals did not indicate any remaining independent factors, providing evidence of unidimensionality. Collectively, these findings supported the use of MFRM analysis.

**Table 1:**
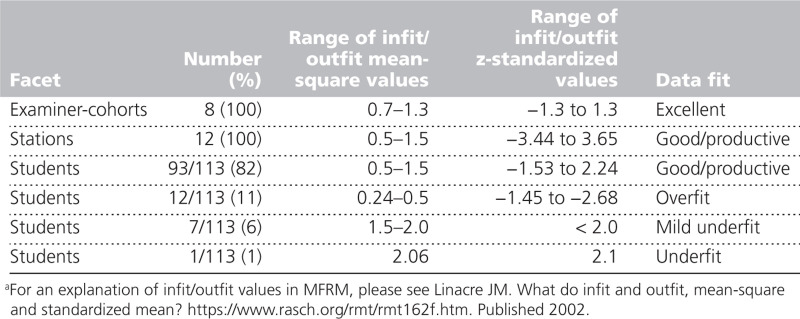
Fit of Data Within Each Facet to the Many Facet Rasch Model (MFRM)^a^

### Influence of examiner-cohorts on students’ scores

**Figure 3:**
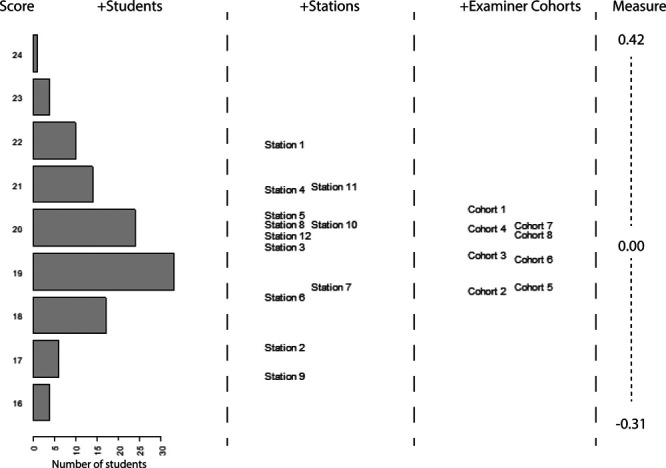
Wright Map showing the relative ability/difficulty/stringency of the facets of, respectively, student, station, and examiner-cohorts. Horizontal bars in the students’ column indicate the number of students at each level of ability. Examiner-cohorts indicate the score that different examiner-cohorts would give to a student of the same ability.

The relative influences of the 3 modeled facets (students, stations, examiner-cohorts) are shown in Figure 3. Adjusted “fair-scores” showed that stations varied in difficulty from 16.6 of 27 (61.5%) to 21.9 (81.1%). As all students performed on all stations, this variability had no systematic influence on students’ scores. After adjusting for the ability of the students whom each group of examiners encountered, “fair-scores” for examiner-cohorts varied between examiner-cohorts from 18.6 of 27 (68.8%) for examiner-cohort 2 (the most stringent or “hawkish” examiner-cohort) to 20.5 (75.9%) for examiner-cohort 1 (the most lenient or “doveish” examiner-cohort). These fair scores can be interpreted as the average scores these groups of examiners would have given to students of the same ability. Since the SD of student ability was 1.43, this difference represents a Cohen’s *d* = 1.3, a large effect.

Adjusting students’ scores for the influence of their examiner-cohorts produced substantial changes for many students: the mean score adjustment was 0.58 points (2%), but 45 students of the 113 (39.8%) had a score adjustment ≥ 0.72 score points (Cohen’s *d* ≥ 0.5), and the largest adjustment was 1.03 score points (3.8%, Cohen’s *d* = 0.72). The number (and percentage) of students who were reclassified ranged from 1 student (0.9% of 113) at a cut score of 16.0 of 27 (59.3%) to 18 students (15.9%) at a cut score of 18.7 (69.3%). The median number of reclassified students was 6 (5.3% of 113), which occurred at a cut score of 17.2 (63.7%).

### Comparison of Internet vs embedded video scoring methods

Examiner participation rates varied by scoring modalities: 43 (90%) of the examiners invited to score embedded videos participated compared with 30 (63%) of the examiners invited to score videos via the Internet. We detected no statistically significant difference between embedded performance scores (mean = 19.02, SD = 4.10) vs Internet performance scores (mean = 19.15, SD = 4.11); the adjusted difference was 0.50 score points (95% CI −0.48 to 1.48, *t* = 1.229(270), *P* = .22). Similarly, we detected no statistically significant difference in the variance of residuals via either modality (embedded 3.03; Internet 3.47, F = 0.763(167, 119), *P* = .108), suggesting the extent of examiner variability did not differ across scoring modalities. In the Internet condition, no significant relationship existed between the MAE for individual examiners’ video scores (i.e., the extent of examiners’ inaccuracy, compared with the mean for that video) and the elapsed time since the OSCE (β = 0.00 [95% CI −0.003 to 0.004], *t* = 0.14, R = 0.013, R^2^ < 0.001, *P* = .88), suggesting that examiners’ scoring did not become more variable over the 3-week period they were allowed to score Internet-based videos.

## Discussion

### Summary of findings

We have replicated the findings of Yeates and colleagues’ 2019 study, ^15^ again showing not only that the difference among examiner-cohorts can be large in some instances (Cohen’s *d* = 1.3) but also that the difference could result in a change in pass/fail outcomes for as many as 16% of students. A range of data supports the VESCA method: video–live score comparisons, scale parameters, dimensionality, and fit statistics. Internet-based video scores were similar to embedded scores, which means Internet-based scoring could potentially offer a more flexible means to facilitate scoring—albeit at some cost in terms of examiner participation rates.

### Implications of findings

The difference we have observed between the highest and lowest scoring examiner-cohorts is consistent with the upper end of estimates described both by Sebok and colleagues ^13^ and by Floreck and Champlain. ^12^ Additionally, this difference is larger than the influences of many of the previously reported influences on assessors’ judgments, ^16,22,23^ including examiner training. ^24–26^ The implications of these differences for the validity of OSCEs depend on the assessments’ purpose ^9^: examiner-cohort effects might affect only a tiny proportion of candidates in an OSCE with a low failure rate used for only pass/fail decisions, whereas a significant minority of candidates may be disadvantaged in an OSCE with a 30% failure rate that is used to rank candidates.

Critical to the interpretation of these findings is whether differences among examiner-cohorts are viewed as a random or systematic influence. If random, then a longer OSCE with greater reliability may ameliorate the observed differences; if systematic, then differences would persist even within a more reliable exam. Given known variations in national standard setting for knowledge testing items ^27^ and suggested differences across sites in large-scale performance-based exams, ^12,13^ there is reason to expect that systematic differences may occur across sites in large-scale exams. These differences would therefore persist in otherwise highly reliable OSCEs.

We suggest that routine measurement of examiner-cohort effects is needed in large-scale OSCEs. Whilst methods based on differential rater functioning ^28^ give valuable insights into some examiner biases, they are unlikely to be informative for fully nested OSCE designs. VESCA promises a feasible means of studying examiner-cohorts in fully nested OSCEs without assuming examiners are stable entities over long time intervals ^29^ or across different stations. ^30^ Given the extent of known examiner variability, ^31^ the medical education community must further examine and discuss the merits of score adjustment based on psychometric analyses.

Embedded and Internet methods of video-scoring by examiners produced similar scores for videos. Embedded scoring produced considerable resource demands: videos had to be processed and made available quickly, and numerous assistants were required to supply examiners with the correct tablets at the right times. Additionally, the processes required for embedded scoring made the afternoon session longer by 40 minutes. Given these constraints, Internet scoring may be more realistic within usual practice.

### Limitations

Despite the rigor of our study, it has some limitations. Modeling relied on the linkage provided by 4 videos per participating examiner (double the number used by Yeates and colleagues in 2019 ^15^), and the linkage of the 4 videos resulted in 17.7% linkage in the data. Whilst more than double the 8% minimum linkage required according to Linacre, ^32^ we recommend caution in interpreting our findings since Linacre’s method optimally balanced the design whilst our method extrapolated from partial crossing. That is, greater linkage might potentially have produced different estimates.

MFRM shows only consistent differences among examiners; if examiners’ scoring was influenced by rater drift, ^33,34^ contrast effects, ^35,36^ examiner–student interactions, ^37^ or idiosyncrasy, ^38^ then these effects would not be adjusted. Fit of data to the model was generally good, but dependability of score adjustment would be limited for students who fit the model less well. Since examiners were not randomized to embedded vs Internet scoring, we cannot exclude the possibility that a difference due to modality was obscured by an unknown confounding effect.

### Future research

Further research, both empirical and using simulation, is needed to determine the following: the accuracy of adjusted scores, how examiners’ stability influences modeling, and the likely effect of operational variables (e.g., the number of linking videos, the choice of statistical analysis method). Research should investigate whether the demographic characteristics of students in the videos (including age, sex, race/ethnicity) influence examiners’ scoring. VESCA constitutes a complex intervention; qualitative exploration should explore stakeholders’ reactions, behaviors, preferences, and trust in VESCA before it is implemented within existing assessment culture.

## Conclusions

Score differences among examiner-cohorts appear to be a significant and replicable effect. Routine consideration should be given to these effects in distributed OSCEs as part of quality assurance procedures. VESCA offers a promising method for studying examiner-cohorts, which could enhance the validity and trust in distributed or national exams.

## Acknowledgments:

The authors offer many thanks to Kirsty Hartley, Sharon Simpson, and the assessments team at the School of Medicine, Keele University; to all the students, examiners, and simulated and volunteer patients who took part in the study; and to the School of Medicine, Keele University for allowing and facilitating the study.

## Supplementary Material


