# Supplementary material for: Measuring the Effect of Examiner Variability in a Multiple-Circuit Objective Structured Clinical Examination (OSCE)
Source: Acad Med. 2021 Mar 2;96(8):1189–96. doi: 10.1097/ACM.0000000000004028 (PMC8300845; doi:10.1097/ACM.0000000000004028)
Supplement: Supplementary file 1 [file acm-96-1189-s001.pdf]

Supplemental Digital Appendix 1

Example of MARKING AND FEEDBACK CATEGORIES from 1 station

N.B. Domains varied by station. The 5 most appropriate domains for each station were selected by station authors.

This example is from a consultation-focused station. Examiners were required to give a mark 1-4 for each of the listed domains.

Please mark the skill categories from 1 – 4:
1 – Must improve in this category
2 – Borderline in this category
3 – Proficient in this category
4 – Very good in this category

NB: station specific examples of the content of each domain were provided for examiners in addition to the generic domain descriptors. These have been excluded due to restrictions on sharing of sensitive examination content by the host institution

Generic domain descriptors:

**History Process**
➢ Enables patient to fully elaborate presenting problem(s)<br>➢ Skilled use of questioning including open and closed questions<br>➢ Clarifies words used and/or symptoms presented by patient as appropriate<br>➢ Recognises and responds appropriately to verbal and non-verbal cues<br>➢ Listens attentively
**History Content**
➢ Obtains sequence of events<br>➢ Obtains details of symptoms<br>➢ Obtains effect on the patient's life<br>➢ Obtains patient's ideas, concerns and expectations<br>➢ Obtains relevant background information including: Past Medical, Drug, Family and Social History; Systems review; Factors influencing health
**Clinical Reasoning**
➢ Seeks relevant and specific information from patient's record or third parties<br>➢ Generates appropriate working diagnoses or problem list<br>➢ Seeks discriminating information from history, examination and investigations to help confirm or refute working diagnoses<br>➢ Correctly interprets information obtained<br>➢ Applies basic, behavioural and clinical sciences to solution of patient's problem<br>➢ Recognises limits of competence and acts accordingly
**Management Content**
➢ Investigates appropriately<br>➢ Refers appropriately
**Building and Maintaining the Relationship**
➢ Develops and maintains a professional relationship with patient<br>➢ Respects the patient's ideas, beliefs and autonomy<br>➢ Responds empathically<br>➢ Fosters collaboration

Examiners were additionally required to provide an overall global score for the performance.

Unedited content. Copyright © by the Association of American Medical Colleges. Unauthorized reproduction is prohibited.

Global Score Descriptors:

**DESCRIPTOR**	
Demonstrates inadequacies in all categories, and one or more serious defects. *Practice likely to result in harm to the patient or self or other staff*	**1**
Demonstrates inadequacies in many categories, and one or more serious defects. *Practice could result in harm to the patient or self or other staff*	**2**
Demonstrates inadequacies in several categories. No serious defects. *Practice unlikely to result in harm to the patient, self or other staff*	**3**
On balance, capable in several relevant categories to a satisfactory standard. Any deficiencies are minor *Competent and Safe practice*	**4**
On balance, capable in many relevant categories to a satisfactory standard. Any deficiencies are minor *Competent and Safe practice*	**5**
Capable in all relevant categories to a high standard. *Skills and attitude are above the level expected for student in this module*	**6**
Capable in all categories to a very high standard. *Skills and attitude are well above the level expected for student in this module*	**7**

Based on these domains, the tablet-based mark system required that examiners gave each candidate a score in each domain:

Domain 1: History Process	1	2	3	4			
Domain 2: History Content	1	2	3	4			
Domain 3: Clinical Reasoning	1	2	3	4			
Domain 4: Management Content	1	2	3	4			
Domain 5: Building and Maintaining the Relationship	1	2	3	4			
Global Judgement:	1	2	3	4	5	6	7

The score on each domain plus the global judgment rating were summed to give a score out of 27.

Unedited content. Copyright © by the Association of American Medical Colleges. Unauthorized reproduction is prohibited.
